# Single-cell transcriptomics reveals expansion of cytotoxic CD4 T cells in supercentenarians

**DOI:** 10.1073/pnas.1907883116

**Published:** 2019-11-12

**Authors:** Kosuke Hashimoto, Tsukasa Kouno, Tomokatsu Ikawa, Norihito Hayatsu, Yurina Miyajima, Haruka Yabukami, Tommy Terooatea, Takashi Sasaki, Takahiro Suzuki, Matthew Valentine, Giovanni Pascarella, Yasushi Okazaki, Harukazu Suzuki, Jay W. Shin, Aki Minoda, Ichiro Taniuchi, Hideyuki Okano, Yasumichi Arai, Nobuyoshi Hirose, Piero Carninci

**Affiliations:** ^a^RIKEN Center for Integrative Medical Sciences, Yokohama, Kanagawa, Japan 230-0045;; ^b^Centre for Supercentenarian Medical Research, Keio University School of Medicine, Tokyo, Japan 160-8582

**Keywords:** centenarian, single-cell transcriptome, CD4 CTL, aging

## Abstract

Supercentenarians, people who have reached 110 y of age, are a great model of healthy aging. Their characteristics of delayed onset of age-related diseases and compression of morbidity imply that their immune system remains functional. Here we performed single-cell transcriptome analysis of 61,202 peripheral blood mononuclear cells (PBMCs), derived from 7 supercentenarians and 5 younger controls. We identified a marked increase of cytotoxic CD4 T cells (CD4 cytotoxic T lymphocytes [CTLs]) as a signature of supercentenarians. Furthermore, single-cell T cell receptor sequencing of 2 supercentenarians revealed that CD4 CTLs had accumulated through massive clonal expansion, with the most frequent clonotypes accounting for 15 to 35% of the entire CD4 T cell population. The CD4 CTLs exhibited substantial heterogeneity in their degree of cytotoxicity as well as a nearly identical transcriptome to that of CD8 CTLs. This indicates that CD4 CTLs utilize the transcriptional program of the CD8 lineage while retaining CD4 expression. Indeed, CD4 CTLs extracted from supercentenarians produced IFN-γ and TNF-α upon ex vivo stimulation. Our study reveals that supercentenarians have unique characteristics in their circulating lymphocytes, which may represent an essential adaptation to achieve exceptional longevity by sustaining immune responses to infections and diseases.

## Significance

Exceptionally long-lived people such as supercentenarians tend to spend their entire lives in good health, implying that their immune system remains active to protect against infections and tumors. However, their immunological condition has been largely unexplored. We profiled thousands of circulating immune cells from supercentenarians at single-cell resolution and identified CD4 T cells that have cytotoxic features. This characteristic is very unique to supercentenarians, because generally CD4 T cells have helper, but not cytotoxic, functions under physiological conditions. We further profiled their T cell receptors and revealed that the cytotoxic CD4 T cells were accumulated through clonal expansion. The conversion of helper CD4 T cells to a cytotoxic variety might be an adaptation to the late stage of aging.

Supercentenarians are rare individuals who reach 110 y of age. They are endowed with high resistance to lethal diseases such as cancer, stroke, and cardiovascular disease (1–4). Demographers in Canada estimated that the chance of living more than 110 y is as low as 1 in 100,000 (http://www.forum.umontreal.ca/forum_express/pages_a/demo.htm). According to the population census covering the whole territory of Japan in 2015 (http://www.stat.go.jp/english/data/kokusei/2015/pdf/outline.pdf), the number of centenarians was 61,763, of which only 146 were supercentenarians. A distinctive feature of supercentenarians is a long healthy lifespan, maintaining relatively high cognitive function and physical independence even after 100 y of age (5, 6). In other words, many supercentenarians can spend almost their entire lives in good health due to the delayed onset of age-related diseases and compression of morbidity (7). Therefore, supercentenarians can be considered a good model of successful aging, and understanding their attributes would be beneficial for superaging societies.

Many functions of the immune system show a progressive decline with age, a phenomenon known as immunosenescence, leading to a higher risk of infection, cancer, and autoimmune diseases (8, 9). A low level of inflammation is the best predictor of successful aging at extreme old age, indicating the importance of maintaining the immune system (10). Age-related alterations are apparent in 2 primary lymphoid organs, thymus and bone marrow, which are responsible for the development of mature lymphocytes (11). In particular, elderly hematopoietic stem cells in bone marrow exhibit a myeloid-biased differentiation potential (12, 13), which causes changes in the cell population of peripheral blood.

Numerous studies have examined age-related alterations in whole blood and peripheral blood mononuclear cells (PBMCs), derived from healthy donors in a wide range of age groups. Fluorescence activated cell sorting (FACS) and transcriptome sequencing technologies, which are extensively used to profile circulating immune cells, have revealed that the population makeup and expression levels of peripheral lymphocytes change dynamically with age. For example, the absolute number and percentage of peripheral blood CD19 B cells decrease with age (14–16). Naïve T cell numbers tend to decrease according to age, whereas antigen-experienced memory T cell numbers increase with concomitant loss of costimulation factors CD27 and CD28 (17). This tendency is more pronounced for CD8 T cells in cytomegalovirus seropositive donors (18). In parallel, transcriptome studies have reported a large number of age-associated genes in bulk peripheral blood that can be used to predict “transcriptomic age” (19). However, most of the studies targeted donors from young to 100 y old, and the circulating immune cells in supercentenarians remain largely unexplored.

Single-cell transcriptomic methods have rapidly evolved in recent years. The accuracy of quantifying gene expression and the number of cells captured per experiment have been dramatically improved (20, 21). These methods have been applied to various subjects such as finding signatures of aging in the human pancreas (22), observing infiltrating T cells in tumors (23, 24), and characterizing diversity of cell types during brain development (25). Here we profiled circulating immune cells in supercentenarians at single-cell resolution and identified unique signatures in supercentenarians that could characterize healthy aging.

## Results

### Single-Cell Transcriptome Profiling of PBMCs.

**Fig. 1.**
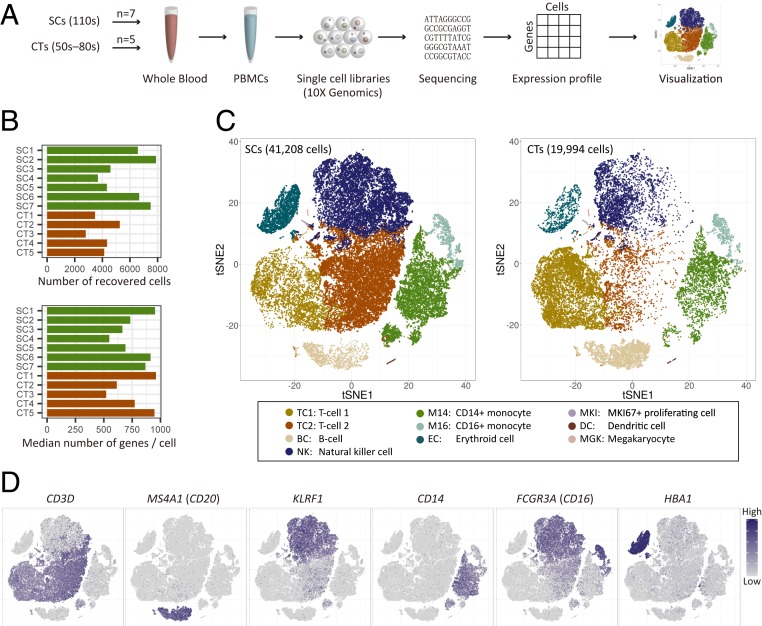
Single-cell transcriptome profiling of PBMCs of supercentenarians and controls. (*A*) Schematic representation of single-cell transcriptome experiments, from blood sample collection to visualization. (*B*) The number of recovered cells that passed quality control and the median number of genes per cell for each of the donors (7 supercentenarians, SC1−SC7; and 5 controls, CT1–CT5). (*C*) Two-dimensional tSNE visualization of PBMCs for supercentenarians (*Left*) and controls (*Right*). Different colors represent 10 clusters (cell types) defined by the *k*-means clustering algorithm. (*D*) Expression of marker genes for 6 major cell types; cell positions are from the tSNE plot in *C*.

We profiled fresh PBMCs derived from 7 supercentenarians (SC1–SC7) and 5 controls (CT1–CT5, aged in their 50s to 80s) by using droplet-based single-cell RNA sequencing technology (10× Genomics) (26, 27) (Fig. 1*A* and *SI Appendix*, Fig. S1*A*). The total number of recovered cells was 61,202 comprising 41,208 cells for supercentenarians (mean: 5,887 cells) and 19,994 cells for controls (mean: 3,999 cells), which is in the normal range of median gene and unique molecular identifier (UMI) counts per cell reported in the 10XQC database (http://10xqc.com/index.html) (Fig. 1*B* and *SI Appendix*, Fig. S1*B*). Based on their expression profiles, we visualized the cells in 2D space using t-distributed stochastic neighbor embedding (tSNE), a method for nonlinear dimensionality reduction. Using a *k*-means clustering algorithm, we found 10 distinct clusters representing different cell types (Fig. 1*C* and *SI Appendix*, Fig. S1 *C* and *D*). We identified the major cell types comprising PBMCs, including: T cells (TC1 and TC2 clusters) characterized by *CD3* and T cell receptor (*TRAC*) expression; B cells (BC cluster) characterized by *MS4A1* (*CD20*) and *CD19* expression; natural killer cells (NK cluster) characterized by *KLRF1* expression; 2 subsets of monocytes (M14 and M16 clusters) characterized by *CD14* and *FCGR3A* (*CD16*) expression, respectively; and erythrocytes (EC cluster) characterized by *HBA1* (hemoglobin alpha locus 1) expression (Fig. 1*D* and *SI Appendix*, Fig. S1*E*). We also found 3 small clusters, annotated as MKI67^+^ proliferating cells (MKI, marker of proliferation Ki-67 positive), dendritic cells (DCs), and megakaryocytes (MGKs), based on the expression of established marker genes (*SI Appendix*, Fig. S1*F*). Although there are some batch effects leading to local enrichment of specific libraries on tSNE plots (*SI Appendix*, Fig. S1*D*), all of the 10 clusters are not library specific, but consisted of cells from more than 11 different donors.

### Significant Reduction of B Cells.

**Fig. 2.**
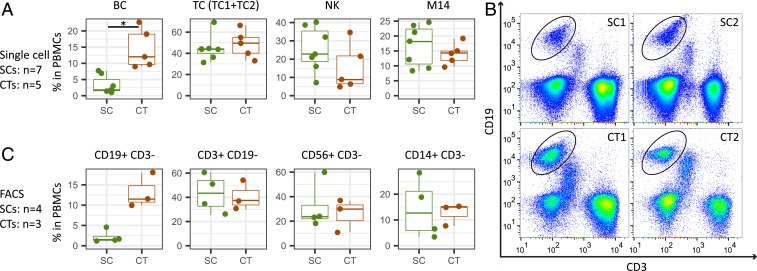
Significant reduction of B cells in supercentenarians. (*A*) Boxplots of the percentage of each cell type (defined by single-cell RNA-Seq) in PBMCs of 7 supercentenarians (SC1–SC7) and 5 controls (CT1–CT5)—the boxes extend from the 25th to 75th percentile and encompass the median (horizontal line). BC, B cell; TC, T cell; NK, natural killer cell; M14, CD14+ monocyte. *P < 0.05 (Wilcoxon rank sum test); no asterisk means not significant. (*B*) Representative FACS plots showing CD19^+^ B cells; the plots for other donors are shown in *SI Appendix*, Fig. S2*B*. (*C*) Boxplots of the percentage of each cell type (defined by FACS) in PBMCs of 4 supercentenarians SC1–SC4 and 3 controls CT1–CT3. No asterisk means not significant (Wilcoxon rank sum test).

In previous FACS analyses using cell-surface markers, various age-associated population changes were observed in human PBMCs, such as B cell reduction (15) and loss of naïve CD8 T cells (18). To understand whether supercentenarians follow the common population changes, we compared the percentages of the immune cells in PBMCs between the supercentenarians and controls. Among the identified cell types in our single-cell transcriptome analysis, B cell numbers were significantly decreased in the supercentenarians compared with the controls (*P* = 0.0025, Wilcoxon rank sum test) (Fig. 2*A*). The median percentage of B cells in the 7 supercentenarians (2%) was far below that in the controls (11%) and the reference values reported in a previous cohort study (28); in contrast, the populations of the other cell types were relatively stable and did not significantly change compared with the controls (Fig. 2*A* and *SI Appendix*, Fig. S2*A*). The reduction of B cells was validated by FACS analysis of 4 supercentenarians (SC1–SC4) and 3 controls (CT1–CT3), which showed low levels of CD3^−^ and CD19^+^ B cell populations in supercentenarians (Fig. 2*B* and *SI Appendix*, Fig. S2*B*). We also confirmed that the percentages of major cell types (B cells, T cells, natural killer cells, and CD14^+^ monocytes) in PBMCs were consistent with those measured by FACS using canonical markers (Fig. 2*C* and *SI Appendix*, Fig. S2*B*). We further clustered the B cells into 3 distinct subtypes (BC1, BC2, and BC3) by using *k*-means clustering (*SI Appendix*, Fig. S2*C*). BC1 corresponds to naïve B cells due to the presence of *IGHD*, an Ig isotype expressed before class switching, and absence of the activation marker *CD27*. BC2 corresponds to quiescent memory B cells, characterized by expression of *CD27*, *IGHG1*, and *IGHA1* (*SI Appendix*, Fig. S2*D*). BC3, which accounts for a small fraction, albeit one with contributions from all donors, shows distinct features of plasma cells such as high levels of immunoglobulins (*IGHA* and *IGHG*), expression of *CD38*, and loss of *MS4A1* (*CD20*) (*SI Appendix*, Fig. S2 *D* and *E*). Among these 3 B cell subtypes in PBMCs, the percentage of naïve B cells was significantly lower in supercentenarians compared with the controls (*P* = 0.005, Wilcoxon rank sum test), and the percentage of memory B cells also tended to be lower in supercentenarians but the difference was not significant (*P* = 0.073) (*SI Appendix*, Fig. S2*F*).

### Expansion of Cytotoxic T Cells in Supercentenarians.

**Fig. 3.**
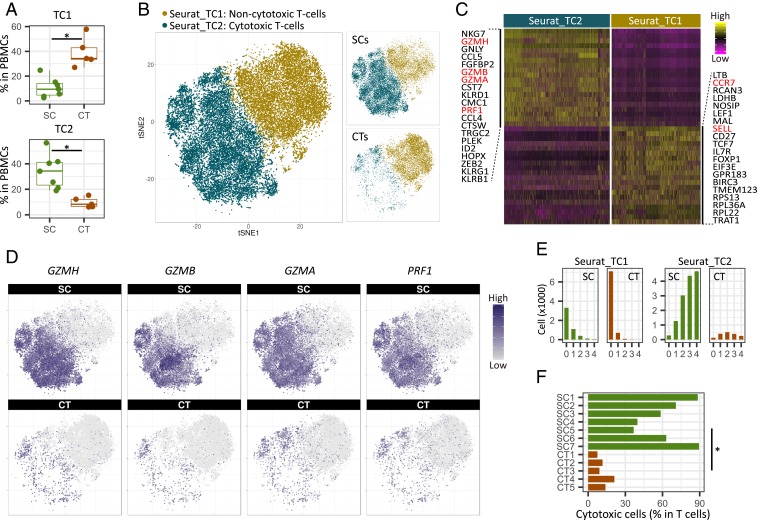
Expansion of cytotoxic T cells in supercentenarians. (*A*) Boxplots of percentages of TC1 and TC2 T cells (defined by *k*-means clustering of single cell RNA-Seq data) in PBMCs of 7 supercentenarians (SC1–SC7) and 5 controls (CT1–CT5). *P < 0.05 (Wilcoxon rank sum test). (*B*) Two-dimensional tSNE visualization of T cells using the Seurat R package. Different colors represent 2 clusters (Seurat_TC1 and Seurat_TC2), similar to the original TC1 and TC2 clusters. *Right* (*Top* and *Bottom*) show supercentenarians and controls, respectively. (*C*) Top 20 genes significantly highly expressed in Seurat_TC2 (*Left*) and Seurat_TC1 (*Right*). Major cytotoxic effector genes and lymph node homing markers are shown in red. (*D*) Expression of cytotoxic genes in supercentenarians (*Top*) and controls (*Bottom*); cell positions are from the tSNE plot in *B*. (*E*) Number of detected genes out of 4 cytotoxic genes (*GZMH*, *GZMB*, *GZMA*, and *PRF1*) per cell. (*F*) Percentage of cytotoxic T cells (cells clustered in TC2) among the total T cells. *P < 0.05 (Wilcoxon rank sum test).

In contrast to the profound reduction of B cells, the T cell fraction remained stable at around 40% of PBMCs according to both the transcriptome data (TC in Fig. 2*A*) and the FACS analysis (CD3^+^CD19^−^ in Fig. 2*C*). However, 2 T cell clusters, TC1 and TC2, were imbalanced between supercentenarians and controls: TC1 was significantly diminished (*P* = 0.0025, Wilcoxon rank sum test), whereas TC2 was significantly expanded (*P* = 0.0025) in supercentenarians (Fig. 3*A*). To better understand this T cell-specific population shift, we extracted all of the cells from TC1 and TC2 for further analysis using the Seurat R package (version 2.3.0) (29). A clustering algorithm based on shared nearest neighbor modularity optimization implemented in Seurat produced 2 major clusters: Seurat_TC1 and Seurat_TC2, corresponding to the original TC1 and TC2 clusters (Fig. 3*B* and *SI Appendix*, Fig. S3*A*). We then compared these 2 clusters and identified 332 differentially expressed genes, of which the most significant gene distinctively expressed in Seurat_TC2 was *NKG7*, a component of granules in cytotoxic lymphocytes. In addition, the top 20 most significant genes included multiple genes encoding cytotoxic effector molecules responsible for the perforin/granzyme apoptosis pathway, such as *GZMH*, *GZMB*, *GZMA*, and *PRF1* (Fig. 3*C* and *SI Appendix*, Fig. S3*B*). In contrast, Seurat_TC1 was characterized by expression of *CCR7* and *SELL* (encoding CD62L), which are required for lymph node migration (*SI Appendix*, Fig. S3*C*). These genes are normally expressed in naïve and central memory T cells, but not in cytotoxic effector memory T cells (30), indicating that the primary factor separating the 2 clusters is cytotoxicity. Perforin/granzyme^+^ cells were predominantly found in the supercentenarians (Fig. 3*D*), whereas CCR7^+^ noncytotoxic cells were more abundant in the controls (*SI Appendix*, Fig. S3*D*). We then examined how many of the 4 cytotoxic genes (*GZMH*, *GZMB*, *GZMA*, and *PRF1*) showed detectable expression in each single cell. As expected, for both the supercentenarians and controls, the vast majority of cells in the noncytotoxic cluster (Seurat_TC1) expressed either 0 or 1 cytotoxic gene(s) (Fig. 3 *E*, *Left*). In the cytotoxic cluster (Seurat_TC2), cells that expressed all 4 genes were abundant in supercentenarians but rare in controls, indicating that the level of cytotoxicity per cell might be higher in supercentenarians (Fig. 3 *E*, *Right*). Cytotoxic T cells were significantly expanded in supercentenarians (*P* = 0.0025, Wilcoxon rank sum test), reaching 80% of T cells in some individuals (Fig. 3*F*). This was in sharp contrast to controls where cytotoxic T cells made up ∼10 to 20% of the total T cell population.

### Expansion of Cytotoxic CD4 T Cells in Supercentenarians.

**Fig. 4.**
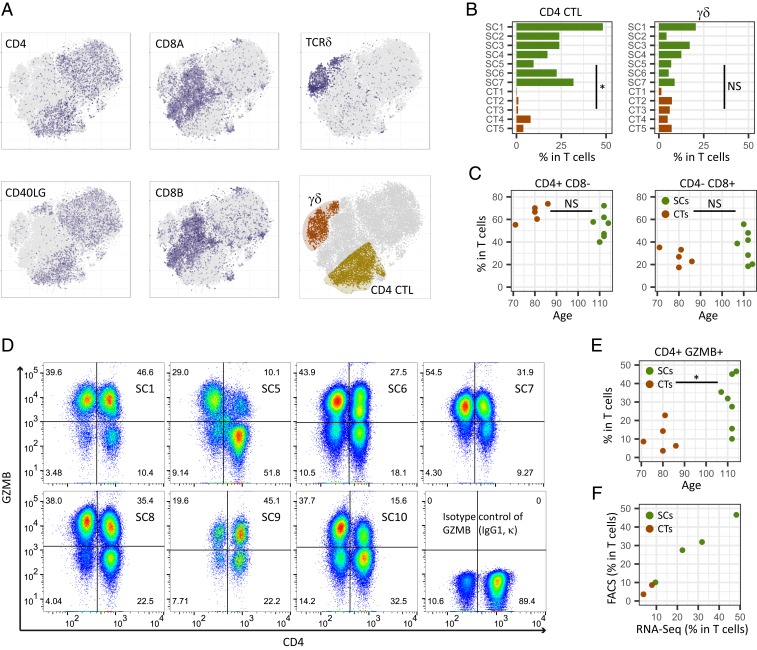
Expansion of cytotoxic CD4 T cells in supercentenarians. (*A*) Classification of cytotoxic T cells into 3 subtypes—CD4 CTLs, CD8 CTLs, and γδ T cells—was based on the expression of *CD4*, *CD8,* and *TRDC* (see also *SI Appendix*, Fig. S4*A*) in T cells of 7 supercentenarians (SC1–SC7) and 5 controls (CT1–CT5); cell positions are from the tSNE plot in Fig. 3*B*. (*B*) Percentages of CD4 CTLs and γδ T cells among the total T cells. *P < 0.05 (Wilcoxon rank sum test); NS, not significant. (*C*) Percentages of CD4^+^ T cells and CD8^+^ T cells in total T cells. NS, not significant (Wilcoxon rank sum test). (*D*) FACS profiles of 6 supercentenarians (SC1, SC5–7, and SC9) and 1 semisupercentenarian (SC8). Cells gated on CD3^+^ were profiled using CD4 (*x* axis) and GZMB or IgG1 κ as an isotype control (*y* axis). Cells in *Top Right* corners are CD4 CTLs. (*E*) Percentages of CD4^+^ GZMB^+^ cells among the total T cells of the 6 supercentenarians and 1 semisupercentenarian listed in *D* and 5 controls (CT4, CT5, and CT6–CT8). *P < 0.05 (Wilcoxon rank sum test). (*F*) Correlation between percentages of CD4 CTLs determined by RNA-Seq and FACS measurements. Each dot represents 1 donor, shown in green for supercentenarians (SC1, SC5–SC7) and red for controls (CT4, CT5).

In general, cytotoxic T cells are CD8^+^ and noncytotoxic helper T cells are CD4^+^, with both being derived from double positive thymocytes (31). Therefore, a simple interpretation of our results is that there is an increase in CD8^+^ T cells in supercentenarians. However, *CD8A* and *CD8B*, which encode the 2 components of CD8, were expressed only in a subset of cytotoxic T cells, whereas *CD4* and *TRDC* (T cell receptor delta constant) were expressed in the other subsets, suggesting the presence of 3 subsets of cytotoxic T cells: CD8 cytotoxic T lymphocytes (CTLs), CD4 CTLs, and γδ T cells (Fig. 4*A*). To investigate cytotoxic T cells other than CD8 CTLs, we manually defined CD4 CTLs and γδ T cells based on ranges of *CD4*, *CD8*, and *TRDC* expression (Fig. 4 *A*, *Bottom Right* and *SI Appendix*, Fig. S4*A*). Previous studies reported that CD4 CTLs account for a tiny fraction of CD4^+^ T cells in PBMCs (e.g., mean 2.2% in 64 healthy donors) (32). Here, the supercentenarians show significantly higher levels of CD4 CTLs (mean, 25.3% of total T cells) than in the controls (mean, 2.8%) (*P* = 0.0025, Wilcoxon rank sum test), as well as higher levels of CD8 CTLs than in the controls (*P* = 0.0025), whereas the population of γδ T cells was moderate in size and comparable to that in the controls (*P* = 0.2) (Fig. 4*B* and *SI Appendix*, Fig. S4*B*). To validate the expansion of CD4 CTLs, we performed FACS analysis of 6 supercentenarians (SC1 and SC5–SC7 [studied above] and SC9 and SC10), 1 semisupercentenarian (over 105 y old; SC8), and 5 controls (CT4 and CT5 [studied above] and CT6–CT8) (*SI Appendix*, Fig. S1*A*) using antibodies against CD3, CD4, CD8, and GZMB. According to the CD4/CD8 staining profile (gated on CD3^+^), the T cells in the supercentenarians were not predominantly CD8^+^ T cells (Fig. 4*C* and *SI Appendix*, Fig. S4*C*). We then asked how many of the CD4^+^ T cells retained in supercentenarians were cytotoxic by using the CD4/GZMB staining profile. Remarkably, CD4^+^GZMB^+^ T cells were quite abundant in the supercentenarians, in which at least 10% (mean, 30.1%) of T cells are CD4 CTLs in all tested supercentenarian samples (*n* = 7) (Fig. 4*D*). The percentages of CD4 CTLs (CD4^+^GZMB^+^ T cells) in the total T cell populations were significantly higher in the centenarians than in the controls (*P* = 0.018, Wilcoxon rank sum test) (Fig. 4*E* and *SI Appendix*, Fig. S4*D*). Furthermore, GZMB^+^ cells were more abundant than GZMB^−^ cells in both CD4 and CD8 T cell populations in 5 out of 7 tested (semi)supercentenarians but none of the controls, indicating expansion of CD4 CTLs as well as CD8 CTLs (*SI Appendix*, Fig. S4*E*). The percentages of CD4 CTLs correlated well between single-cell RNA-Seq and FACS analyses according to the comparison of the 6 commonly analyzed samples (4 supercentenarians and 2 controls) (Fig. 4*F*). Thus, the high level of CD4 CTLs in supercentenarians was supported by 2 independent methods. Finally, we assessed protein levels of 2 cytotoxic molecules, perforin and granulysin, together with granzyme B in 1 of the supercentenarians (SC2) using FACS. According to the GZMB/PRF1 and GZMB/GNLY staining profiles (gated on live CD3^+^ CD4^+^ CD8^−^), the CD4^+^GZMB^+^ T cells were predominantly perforin positive, but not necessarily granulysin positive (*SI Appendix*, Fig. S4*F*), suggesting that the composition of cytotoxic granules might be different in the CD4^+^GZMB^+^ population.

### Limited Numbers of CD4 CTLs in Young Donors.

Our main focus in this study is on the analysis of elderly subjects and supercentenarians, in which young subjects are missing in our cohort. To explore CD4 CTLs in young subjects, we used a publicly available single-cell dataset (33), generated by Chromium Single Cell 3ʹ v2 Reagent Kits, the same kits for our analysis. The dataset profiles cryopreserved PBMCs from 45 donors ranging in age from the 20s to 70s (*SI Appendix*, Fig. S5*A*). We downloaded the gene expression matrix (UMI counts) for 18,233 T cells (median: 377 T cells per donor) (*SI Appendix*, Fig. S5*B*). We confirmed that *CD3* genes are expressed in the vast majority of the cells, a subset of which express *CD4* or *CD8* genes (*SI Appendix*, Fig. S5*C*). Our tSNE plot based on the expression profile consistently separated CD4 and CD8 T cells, defined by the authors in the original paper (33) (*SI Appendix*, Fig. S5*D*). We found that expressions of cytotoxic genes (*GZMH*, *GZMB*, *GZMA*, and *PRF1*) are highly restricted to the CD8 T cell population, whereas naïve and central memory markers (*CCR7* and *SELL*) are mainly expressed in CD4 T cells (*SI Appendix*, Fig. S5*E*). We further confirmed that expressions of *GZMB* and *PRF1* are rarely expressed in the CD4 population in all age groups (20 to 30s, 40s, 50s, and 60 to 70s) (*SI Appendix*, Fig. S5*F*). Less than 4% of CD4 T cells in all donors have a cytotoxic feature, defined as at least 2 UMI counts from 4 cytotoxic genes (*GZMH*, *GZMB*, *GZMA*, and *PRF1*), indicating limited numbers of CD4 CTLs in young, middle, and old donors up to the 70s with no significant difference between any 2 age groups (*SI Appendix*, Fig. S5*G*).

### Cell State Transition of CD4 CTLs during T Cell Differentiation.

**Fig. 5.**
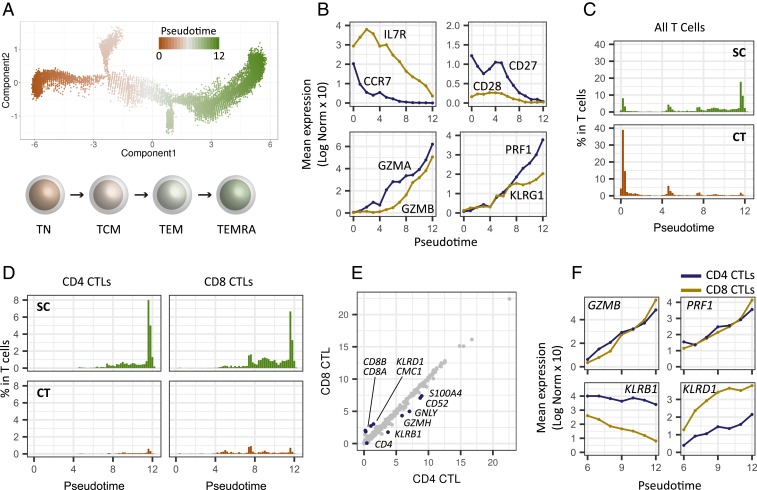
The differentiation state of T cells for 7 supercentenarians (SC1–SC7) and 5 controls (CT1–CT5). (*A*) Pseudotime trajectory of T cells estimated using Monocle 2. A continuous value from 0 to 12 was assigned to each cell as a pseudotime. The *Bottom* shows the general scheme of T cell differentiation. TN, naïve; TCM, central memory; TEM, effector memory; and TEMRA, effector memory reexpressing CD45RA. (*B*) Expression transition of differentiation-associated genes along the pseudotime. (*C*) Percentages of T cells along the pseudotime for supercentenarians (SC) and controls (CT). (*D*) Percentages of CD4 and CT8 CTLs among the total T cells along the pseudotime. (*E*) Correlation of gene expression between CD4 and CD8 CTLs. (*F*) Expression transition of selected genes shown separately for CD4 and CD8 CTLs.

CD4 CTLs have been identified in differentiated T cell subsets, i.e., effector memory (TEM) and effector memory reexpressing CD45RA (TEMRA) cells, which are often associated with a distinct surface phenotype including CCR7^−^, CD27^−^, CD28^−^, and CD11A^+^ (32, 34). To understand the CD4^+^GZMB^+^ T cells in the context of differentiation, we constructed single-cell trajectories using the Monocle 2 (version 2.4.0) R package (35); all T cells in TC1 and TC2 were placed on these trajectories based on changes in their transcriptomes (Fig. 5*A* and *SI Appendix*, Fig. S6*A*). Consistent with the clustering analyses, TC1 (the noncytotoxic cluster) was mostly distributed throughout the early pseudotime, whereas TC2 (the cytotoxic cluster) was found mostly in later pseudotime, showing a clear temporal separation of the 2 (*SI Appendix*, Fig. S6*B*). We then examined the transition of expression values along pseudotime for a panel of established marker genes associated with T cell differentiation (30). As mentioned above, *CCR7* expression is a primary marker of central memory T cells and distinguishes them from effector memory T cells. We observed rapid reduction of *CCR7* expression followed by the gradual loss of costimulatory molecules *CD27* and *CD28* (Fig. 5*B*), indicating that early pseudotime corresponds to naïve and central memory T cells. The results also showed a gradual increase of expression of *GZMA*, *GZMB*, and *PRF1*, which encode cytotoxic molecules, as well as concordant patterns of expression of transcripts encoding adhesion and migration molecules (Fig. 5*B* and *SI Appendix*, Fig. S6*C*), indicating progressive differentiation states of effector memory T cells, corresponding to late pseudotime. One of the branches showed enriched expression of *FOXP3* and *IL2RA* (*CD25*), primary markers of regulatory T cells (*SI Appendix*, Fig. S6 *D* and *E*). Altogether the backbone of pseudotime estimated by Monocle 2 recapitulated T cell differentiation starting from naïve and central memory to terminally differentiated effector memory states with a branched trajectory of regulatory T cell-like features. We examined the distributions of T cells along pseudotime separately for supercentenarians and controls. The T cells of the supercentenarians were clearly shifted toward more differentiated states compared with those of the controls (Fig. 5*C*): nearly 60% of T cells in the controls were placed in the earliest pseudotime corresponding to naïve and central memory T cells, whereas T cells of supercentenarians were enriched in late pseudotime. Next, we examined the distributions of CD4 CTLs (*n* = 5,274) and CD8 CTLs (*n* = 7,643), which were defined in Fig. 4*A* and *SI Appendix*, Fig. S4*A*. CD4 CTLs were distributed in the latter half of pseudotime in a similar way to CD8 CTLs (Fig. 5*D* and *SI Appendix*, Fig. S6*F*), indicating a similar differentiation process despite fundamental functional differences between the 2 cell types. Indeed, mean expression values were highly correlated between CD4 and CD8 CTLs, with the exception of a small number of genes (Fig. 5*E*). The expression of 4 major cytotoxic genes *GZMA*, *GZMB*, *PRF1*, and *NKG7*, which are known to be abundant in CD4 CTLs (32, 36), increased along the latter half of pseudotime in a similar manner between CD4 and CD8 CTLs; however, the expression of 2 other major cytotoxic genes, *GZMH* and *GNLY*, showed slightly different patterns for CD4 and CD8 CTLs (Fig. 5*F* and *SI Appendix*, Fig. S6*G*). Other exceptions were *KLRB1* and *KLRD1,* which encode 2 killer cell lectin-like receptors; at all time points, expression of these genes was higher in either CD4 or CD8 CTLs. In summary, we found a seemingly heterogeneous population of CD4 CTLs, which could be further categorized in pseudotime according to differentiation states. These differentiation states were characterized by progressive transcriptional changes, in a similar fashion to CD8 CTLs.

### Clonal Expansion of CD4 CTLs.

**Fig. 6.**
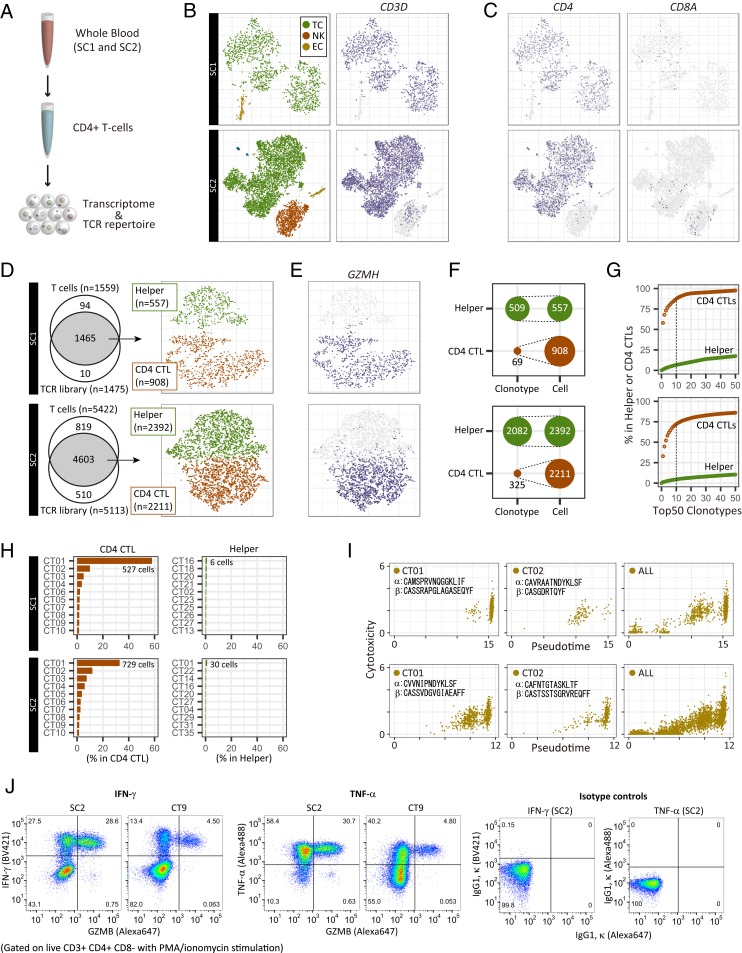
Single-cell transcriptome and TCR profiles of CD4^+^ T cells for 2 supercentenarians (SC1, *Top*; and SC2, *Bottom*). (*A*) Schematic representation of experiments for the single-cell transcriptome and TCR analysis. (*B*) Two-dimensional tSNE visualization of 3 cell types (TC, T cell; NK, natural killer cell; EC, erythroid cell), and CD3D expression (*Right*). (*C*) Expression of marker genes for CD4 and CD8 T cells; cell positions are from the tSNE plot in *B*. (*D*) T cells recovered in both transcriptome and TCR libraries. Recovered cells were clustered into helper T cells and CD4 CTLs, shown in the tSNE plot. (*E*) Expression of a marker gene for cytotoxic T cells; cell positions are from the tSNE plot in *D*. (*F*) Diversity of TCRs in helper T cells and CD4 CTLs. (*G*) Cumulative occupancy of the top 50 most abundant clonotypes. (*H*) Occupancy of the top 10 most abundant clonotypes. (*I*) Pseudotime and cytotoxicity of clonally expanded CD4 T cells. Cytotoxicity values indicate the mean expression of 5 cytotoxic genes: *NKG7*, *GZMA*, *GZMB*, *GZMH*, and *PRF1*. (*J*) FACS profiles of 1 supercentenarian (SC2) and 1 young control (CT9) upon PMA/ionomycin stimulation. Cells gated on live CD3^+^ CD4^+^ CD8^−^ were profiled using GZMB or IgG1 κ (*x* axis) and IFN-γ, TNF-α, or IgG1 κ (*y* axis).

To explore the mechanism by which CD4 CTLs increased in supercentenarians, we performed an integrative analysis of the single-cell transcriptome and the T cell receptor (TCR) repertoire. Firstly, we asked whether the high level of CD4 CTLs was reproducible at a different time point to that studied above. We recollected fresh whole blood samples from 2 supercentenarians (SC1 and SC2) about 1.5 y after the first collection and isolated CD4^+^ T cells by negative selection (Fig. 6*A*). The single-cell transcriptome profile generated using the Seurat R package confirmed high enrichment of T cells, characterized by the expression of *CD3* genes (Fig. 6*B* and *SI Appendix*, Fig. S7*A*). B cells and CD14 monocytes were mostly depleted, whereas natural killer cells and erythroid cells were not completely depleted in the libraries (*SI Appendix*, Fig. S7*B*). In the T cell population, CD4^+^ T cells were strongly enriched and CD8^+^ T cells were depleted (Fig. 6*C* and *SI Appendix*, Fig. S7*C*). We could recover transcripts encoding TCR alpha and beta chains in most of the T cells, which were further clustered into 2 distinct cell types, based on the expression profiles (Fig. 6*D* and *SI Appendix*, Fig. S7*D*). One of the clusters comprised CD4 CTLs, characterized by the coexpression of *GZMH*, *GZMA*, *GZMB*, *NKG7*, and *PRF1* as well as low expression of *SELL*, *CD27*, and *CCR7* (Fig. 6*E* and *SI Appendix*, Fig. S7 *E* and *F*). CD4 CTLs accounted for about 62% (SC1) and 48% (SC2) of the CD4 T cells in this analysis, which is consistent with the first sample collection from the same donor (Fig. 4*B*). This observation indicates that CD4 CTLs of supercentenarians are not transiently accumulated but persist in the blood for years.

Secondly, we assessed the diversity of TCRs in CD4 CTLs and noncytotoxic helper T cells. We defined clonotypes based on CDR3 sequences of both TCR alpha and beta chains using the Cell Ranger analysis pipeline. We identified clonally expanded CD4 CTLs, which have only 69 clonotypes, among 908 cells in SC1 and 325 clonotypes among 2,211 cells in SC2 (Fig. 6*F*). Moreover, the top 10 clonotypes occupied more than 70% of CD4 CTLs but less than 10% of helper T cells (Fig. 6*G*). Both supercentenarians had 1 massively expanded clonotype, “CT01,” which accounted for 15 to 35% of the entire CD4 T cell population (Fig. 6*H*), and had distinct combinations of TCR alpha and beta chains (TRAV12-3/TRAJ23 and TRBV3-1/TRBD2/TRBJ2-7 for SC1 and TRAV12-1/TRAJ20 and TRBV9/TRBD2/TRBJ1-1 for SC2). The cells of both “CT01” and “CT02” were mostly distributed in the CD4 CTL cluster with cytotoxic features (*SI Appendix*, Fig. S7*G*) and were rarely placed in the CD4 helper T cell cluster. The low TCR diversity of CD4 CTLs is in sharp contrast to helper T cells in the same donor as well as younger controls, including publicly available CD4 and CD8 T cells (Fig. 6*H* and *SI Appendix*, Fig. S7*H*). We compared clonotypes (TCR alpha and beta chains) found in SC1 and SC2 with 2 TCR databases, VDJdb (37) and McPAS-TCR (38); however, none of the clonotypes were found in the public databases. We also compared clonotypes between SC1 and SC2, and identical TCRs between 2 supercentenarians were not detected.

To understand the differentiation status of clonally expanded cells, we constructed a single-cell trajectory of CD4 T cells using Monocle 2. The CD4 T cells were distributed along pseudotime, following an increasingly differentiated trajectory, as evidenced by the marker gene expression patterns (*SI Appendix*, Fig. S7*I*). As expected, at the late pseudotime, the top 2 expanded clonotypes CT01 and CT02 were enriched with highly expressed cytotoxic genes (Fig. 6*I* and *SI Appendix*, Fig. S7*I*). Nevertheless, a subset of these cells was found in less differentiated states, indicating that a large number of CD4 T cell clones with the same TCRs but at different levels of differentiation are circulating in the blood.

Finally, we examined whether CD4 CTLs derived from supercentenarians could produce inflammatory cytokines. We stimulated CD4 T cells remaining from the single-cell TCR analysis with phorbol 12-myristate 13-acetate (PMA) and ionomycin in the presence of brefeldin A for 6 h. The cells were analyzed by FACS using antibodies against CD3, CD4, CD8, GZMB, IFN-γ, and TNF-α. According to the GZMB/IFN-γ staining profile (gated on live CD3^+^ CD4^+^ CD8^−^), the vast majority of CD4^+^GZMB^+^ T cells are IFN-γ^+^ in supercentenarian and control samples (Fig. 6*J*). TNF-α is also positive in the same CD4^+^GZMB^+^ population, indicating that CD4 CTLs have ability to produce inflammatory cytokines upon ex vivo stimulation. Note that the FACS data for SC1 also indicates the production of IFN-γ and TNF-α from the CD4^+^GZMB^+^ population, although the number of available CD4 T cells were very small (*SI Appendix*, Fig. S7*J*).

## Discussion

Here, we identified signatures of supercentenarians in circulating lymphocytes by using single-cell transcriptome analyses. In particular, CD4 CTLs were strongly expanded with distinct expression profiles including the activation of *GZMA*, *GZMB*, *GZMH*, *PRF1*, *NKG7* (*TIA-1*), *GNLY*, *CD40LG*, *KLRG1*, *KLRB1*, and *ITGAL* (*CD11A*) and the suppression of *CCR7*, *CD27*, *CD28*, and *IL7R* (Figs. 3*D*, 4*A*, and 5 *B* and *F*). The results of single-cell TCR repertoire analysis of 2 supercentenarians suggest that the cell state transition of CD4 T cells is at least partially explained by clonal expansion due to repeated stimulation with the same antigen. Here we discuss potential functions of CD4 CTLs in the late stage of aging in terms of protective roles against tumor development and viral infections.

The primary function of CD4 T cells, generally called helper T cells, is the regulation of immune responses using various cytokines, rather than direct elimination of target cells using cytotoxic molecules. Nevertheless, the presence of CD4 T cells with cytotoxic features, namely CD4 CTLs, has been repeatedly reported in humans and mice (32, 34, 39). The reported fractions of CD4 CTLs are generally as low as a few percent of the total CD4 T cells in healthy PBMCs (32, 40), whereas the size of the CD4 CTL fraction in the supercentenarians analyzed was on average 25% of T cells, as measured by RNA-Seq and supported by the independent FACS measurements (Fig. 4 *B* and *E*). More intriguingly, 5 out of 7 supercentenarians analyzed by FACS had more GZMB^+^ than GZMB^−^ CD4 T cells (Fig. 4*D*). The physiological role of the expanded CD4 CTLs remains unclear in humans, however a recent single-cell transcriptome study identified tumor-infiltrating CD4 CTLs in human hepatocellular carcinoma (23). In addition, several studies demonstrate that CD4 CTLs have the ability to directly kill tumor cells and eradicate established tumors in an MHC class II-dependent manner in mouse models (41, 42). Importantly, CD8 CTLs recognize class I MHC molecules present in nearly all cells. In contrast CD4 CTLs recognize class II MHC molecules, which are usually absent in normal nonimmune cells, but present in a subset of tumor cells (43). This indicates that CD4 CTLs might contribute tumor immunity against established tumors and may have an important role in immunosurveillance, helping to identify and remove incipient tumor cells abnormally activating class II MHC molecules.

Another potential function of CD4 CTLs is antiviral immunity. A growing number of studies have demonstrated the direct cytotoxic activity, protective roles, and the associated induction of CD4 CTLs against various viruses such as dengue virus, influenza virus, hepatitis virus, CMV (cytomegalovirus), and HIV (44–48). Clonally expanded CD4 CTLs with virus-specific TCRs have been identified in dengue virus-positive donors (40). The association of CD4 CTLs with virus infection suggests that CD4 CTLs have accumulated in supercentenarians at least partially through clonal expansions triggered by repeated viral exposure. Although some important genes such as *CRTAM* and *ADGRG1* (*GPR56*) have been reported (34, 49), the exact molecular mechanism of the conversion from CD4 helper T cells to CD4 CTLs is still unclear. Our transcriptome data show the striking similarity of gene expression and differentiation between CD4 CLTs and CD8 CTLs (Fig. 5 *D* and *E*), suggesting that CD4 CTLs use the CD8 transcriptional program internally, while retaining CD4 expression on the cell surface. Indeed, CD4 CTLs extracted from supercentenarians produced IFN-γ and TNF-α upon ex vivo stimulation (Fig. 6*J*). This agrees with the previous finding that CD4 helper T cells can be reprogrammed into CD4 CTLs by the loss of ThPOK (also known as ZBTB7B), the master regulator of CD4/CD8 lineage commitment, with concomitant activation of CD8 lineage genes (50). The reinforcement of the cytotoxic ability by the conversion of CD4 T cells in supercentenarians might be an adaptation to the late stage of aging, in which the immune system needs to eliminate abnormal or infected cells more frequently. It should be noted, however, that antigens recognized by clonally expanded TCRs are not known, and further work is required to characterize CD4 CTLs in supercentenarians.

## Materials and Methods

### Human Blood Samples.

All experiments using human samples in this study were approved by the Keio University School of Medicine Ethics Committee (approval no. 20021020) and the ethical review committee of RIKEN (approval no. H28-6). Informed consent was obtained from all donors. Fresh whole blood from supercentenarians, their offspring residing with them, and unrelated donors was collected in 2-mL tubes containing ethylene diamine tetraacetic acid (EDTA). PBMCs were isolated from whole blood within 8 h of sample collection by using SepMate-15 tubes (STEMCELL Technologies) with Ficoll-Paque Plus (GE Healthcare Life Sciences) according to the manufacturer’s instructions. Briefly, each blood sample was diluted with an equal volume of PBS plus 2% FBS, added into a SepMate tube, and centrifuged at 1,200 × *g* for 10 min at room temperature. Enriched mononuclear cells were washed with PBS plus 2% FBS and twice centrifuged at 300 × *g* for 8 min. Cell numbers and viability were measured using a Countess II Automated Cell Counter (Thermo Fisher Scientific).

### Single-Cell Library Preparation.

Single-cell libraries were prepared from freshly isolated PBMCs by using Chromium Single Cell 3ʹ v2 Reagent Kits (26). The cells and kit reagents were mixed with gel beads containing barcoded oligonucleotides (UMIs) and oligo dTs (used for reverse transcription of polyadenylated RNAs) to form reaction vesicles called gel bead-in-emulsions (GEMs). The barcoded cDNAs in each GEM were pooled for PCR amplification, and adapter and sample indices were added. Single-cell libraries were sequenced with paired-end reads on the Illumina HiSEq. 2500 platform, with mostly 1 sample per lane. The remaining PBMCs were suspended in CELLBANKER cryopreservation medium (ZENOAQ), and stored at −80 °C.

### Single-Cell Data Processing.

The analysis pipelines in Cell Ranger version 2.1.0 were used for sequencing data processing. FASTQ files were generated using *cellranger mkfastq* with default parameters. Then, *cellranger count* was run with–transcriptome = refdata-cellranger-GRCh38-1.2.0 for each sample, in which reads had been mapped on the human genome (GRCh38/hg38) using STAR (version 2.5.1b) (51) and UMIs were counted for each gene. The outputs of *cellranger count* for individual samples were integrated using *cellranger aggr* with–normalize = mapped, in which read depths are normalized based on the confidently mapped reads. This command also runs principal component analysis (PCA), tSNE, and *k*-means clustering algorithms to visualize clustered cells in 2D space. The output of *cellranger aggr* was loaded into R by using an R package, Cell Ranger R Kit (version 2.0.0), developed by 10× Genomics (http://cf.10xgenomics.com/supp/cell-exp/rkit-install-2.0.0.R). Log-normalized expression values of all annotated genes were calculated using 2 functions, *normalize_barcode_sums_to_median* and *log_gene_bc_matrix*, implemented in the R package.

### Analysis of B Cell Subsets.

Cells categorized in the B cell cluster by the *k*-means clustering were extracted and saved as a file using the *save_cellranger_matrix_h5* function in the R package Cell Ranger R Kit. This file was loaded into *cellranger reanalyze* to rerun PCA, tSNE, and *k*-means (*k* = 3) clustering algorithms. Wilcoxon rank sum test was applied to compare percentages of B cells between the supercentenarians and controls using the wilcox.test function in R.

### Analysis of T Cell Subsets.

The Seurat R package (version 2.3.0) was used to analyze T cell subsets (TC1 and TC2). The outputs of *cellranger count* were loaded using the *Read10X* function. Cells clustered in TC1 and TC2 by the Cell Ranger analysis pipelines were extracted, and principal components were calculated using *RunPCA* function. The first 16 principal components, based on the manual inspection of the elbow plot (*PCElbowPlot*), were used for cell clustering (using the *FindClusters* function with resolution 0.05) and tSNE visualization (using *RunTSNE*). Differentially expressed genes were identified using the *FindAllMarkers* function, and the top 20 genes were visualized in a heatmap using the *DoHeatmap* function. CD4 CTL, CD8 CTL, and γδ T cell clusters were manually defined in the interactive mode of the tSNE plot by using the *TSNEPlot* function with do.identify = TRUE, based on the expression of marker genes. Wilcoxon rank sum test was applied to compare percentages of T cell subtypes between the supercentenarians and controls using the wilcox.test function in R.

### Analysis of T Cells in Young Donors from a Public Dataset.

The single-cell RNA-Seq data of PBMCs derived from 45 healthy donors were generated and released by van der Wijst et al. (33). Freely available UMI count data for T cells were downloaded and visualized in 2D space (tSNE) based on the expression profile using the Seurat R package (version 2.3.0). Age information of individual donors was extracted from Supplementary Table 8 of the original paper (32).

### Pseudotime Analysis.

Monocle 2 (version 2.4.0) was used to estimate a pseudotemporal path of T cell differentiation (35). Cells clustered in TC1 and TC2 by Cell Ranger analysis pipelines were loaded to create a Monocle object using the *newCellDataSet* function implemented in Monocle 2. The cells were ordered in pseudotime along a trajectory using *reduceDimension* with the DDRTree method and *orderCells* functions. Mean log-normalized expression values of selected marker genes were calculated for each bin from 0 to 12 pseudotime points.

### Antibodies and Flow Cytometric Analysis.

Cryopreserved PBMCs were thawed and suspended in FACS buffer (1× Hank’s balanced salt solution with 2% FBS and 0.2% NaN3). Monoclonal antibodies specific for human CD3ε (UCHT1 and HIT3a), CD4 (RPA-T4), CD8 (RPA-T8), CD19 (HIB19), CD14 (M5E2), CD16 (B73.1), CD56 (B159), GzmB (GB11), Perforin (δG9), and Granulysin (RB1), were purchased from BD Pharmingen. Cell numbers were counted using a Countess II Automated Cell Counter. For intracellular staining, cells were fixed and permeabilized with IntraPrep Permeabilization Reagent (Beckman Coulter) according to the manufacturer’s protocols. Cells were analyzed using FACSAria III and FACSAria SORP cell sorters (BD Biosciences) with FlowJo Software (version 10.4.2).

### Single-Cell TCR Analysis.

RosetteSep Human CD4^+^ T Cell Enrichment Mixture with SepMate-15 (STEMCELL Technologies) was used to remove non-CD4^+^ T cells from fresh whole blood. A single-cell transcriptome library was prepared from the enriched CD4^+^ T cells by using the Chromium Single Cell 5ʹ Library Kit (10× Genomics) with 50 ng of cDNA amplified product. A single-cell TCR library was prepared using Chromium Single Cell V(D)J Enrichment Kits, Human (10× Genomics). The libraries were sequenced with paired-end 150-bp reads on the Illumina HiSEq. 2500 platform. Analysis pipelines in Cell Ranger version 3.0.2 (updated version was used for the 5′ single-cell and TCR libraries from version 2.1.0 used for the 3′ single-cell libraries) were used for the sequencing data processing. TCR data were processed by running cellranger vdj with–reference = refdata-cellranger-vdj-GRCh38-alts-ensembl-2.0.0 to assemble TCR alpha and beta chains and determine clonotypes. Transcriptome data were processed by running cellranger count with–transcriptome = refdata-cellranger-GRCh38-1.2.0. The Seurat R package (version 2.3.0) was used for cell clustering (FindClusters) and tSNE visualization (RunTSNE). Three control datasets of T cell clonotypes analyzed by the same 10× Genomics kits were downloaded from the 10× Genomics websites below (a simple registration is needed).

T cells: http://cf.10xgenomics.com/samples/cell-vdj/3.0.0/vdj_v1_hs_pbmc2_t/vdj_v1_hs_pbmc2_t_clonotypes.csv
CD4 T cells: http://cf.10xgenomics.com/samples/cell-vdj/2.2.0/vdj_v1_hs_cd4_t/vdj_v1_hs_cd4_t_clonotypes.csv
CD8 T cells: http://cf.10xgenomics.com/samples/cell-vdj/2.2.0/vdj_v1_hs_cd8_t/vdj_v1_hs_cd8_t_clonotypes.csv

### Cell Culture and Stimulation with PMA and Ionomycin.

Cryopreserved PBMCs and CD4^+^ T cells were thawed at 37 °C and washed once with X-VIVO 20 (Lonza). The cells were centrifuged at 500 × *g* for 5 min, resuspended in X-VIVO 20 at a concentration of 1.0 × 10^6^ cells/mL, and incubated at 37 °C in 5% CO_2_ for 16 h. The cells were stimulated with cell activation mixture containing PMA and ionomycin (BioLegend) and brefeldin A (eBioscience), and incubated for 6 h. The stimulated cells were harvested, washed once with 500 μL of PBS with 2% FBS, and treated with Human BD Fc Block (BD Biosciences). Dead cells were stained with Fixable Viability Dye eFluor 780 (eBioscience). Cell-surface proteins were stained with specific antibodies against CD3ε (UCHT1), CD4 (RPA-T4), and CD8 (RPA-T8). The cells were fixed and permeabilized with Fixation/Permeabilization solution and BD Perm/Wash buffer (BD Biosciences). Intracellular molecules were stained with specific antibodies against GzmB (GB11), IFN-γ (4S.B3), and TNF-α (Mab11) or isotype controls, IgG1 κ (×40), IgG1 κ (MOPC-21), and IgG2b κ (28–36). Cells were analyzed using FACSCanto II (BD Biosciences) with FlowJo Software (version 10.4.2). All FACS antibodies were purchased from BD Pharmingen.

### Statistical Analysis.

The statistical significance of differences between supercentenarians and controls were determined by a 2-sided Wilcoxon rank sum test using the wilcox.test function in R. *P* values are indicated with an asterisk (*P < 0.05) in the figures.

### Data Availability.

Raw UMI counts and normalized expression values for single-cell RNA-Seq are publicly available at http://gerg.gsc.riken.jp/SC2018/. Individual sequencing read data will be available on request under the condition of approval of the ethics committee of Keio University and material transfer agreement.

## Supplementary Material

Supplementary File
