# Supplementary material for: Single-cell transcriptomics reveals expansion of cytotoxic CD4 T cells in supercentenarians
Source: Proc Natl Acad Sci U S A. 2019 Nov 12;116(48):24242–51. doi: 10.1073/pnas.1907883116 (PMC6883788; doi:10.1073/pnas.1907883116)
Supplement: Supplementary File [file pnas.1907883116.sapp.pdf]

Figure S1

A

ID	Age	Sex	Type	RNA-Seq	FACS1	FACS2	Name
SC1	110s	F	-	**O**	O	O	RKNS001
SC2	110s	M	-	**O**	O	O	RKNS002
SC3	110s	F	-	**O**	O	-	RKNS003
SC4	110s	F	-	**O**	O	-	RKNS005
SC5	110s	F	-	**O**	-	O	RKNS007
SC6	110s	F	-	**O**	-	O	RKNS014
SC7	110s	M	-	**O**	-	O	RKNS016
CT1	50s	M	Un	**O**	O	-	RKNS000
CT2	70s	F	Off	**O**	O	-	RKNS004
CT3	60s	F	Un	**O**	O	-	RKNS006
CT4	70s	F	Un	**O**	-	O	RKNS012
CT5	80s	M	Off	**O**	-	O	RKNS013
SC8	100s	M	-	-	-	O	RKNS008
SC9	110s	F	-	-	-	O	RKNS017
SC10	110s	F	-	-	-	O	RKNS011
CT6	80s	M	Off	-	-	O	RKNS009
CT7	80s	F	Un	-	-	O	RKNS010
CT8	80s	M	Off	-	-	O	RKNS015
CT9	40s	M	Un	-	-	O	RKNS000

Off: offspring, Un: unrelated

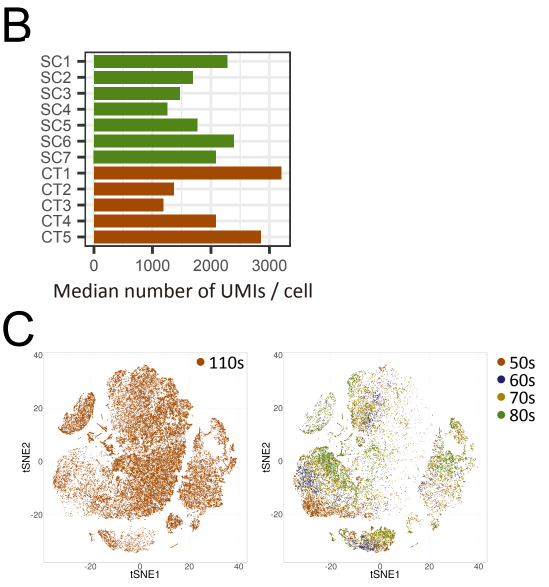

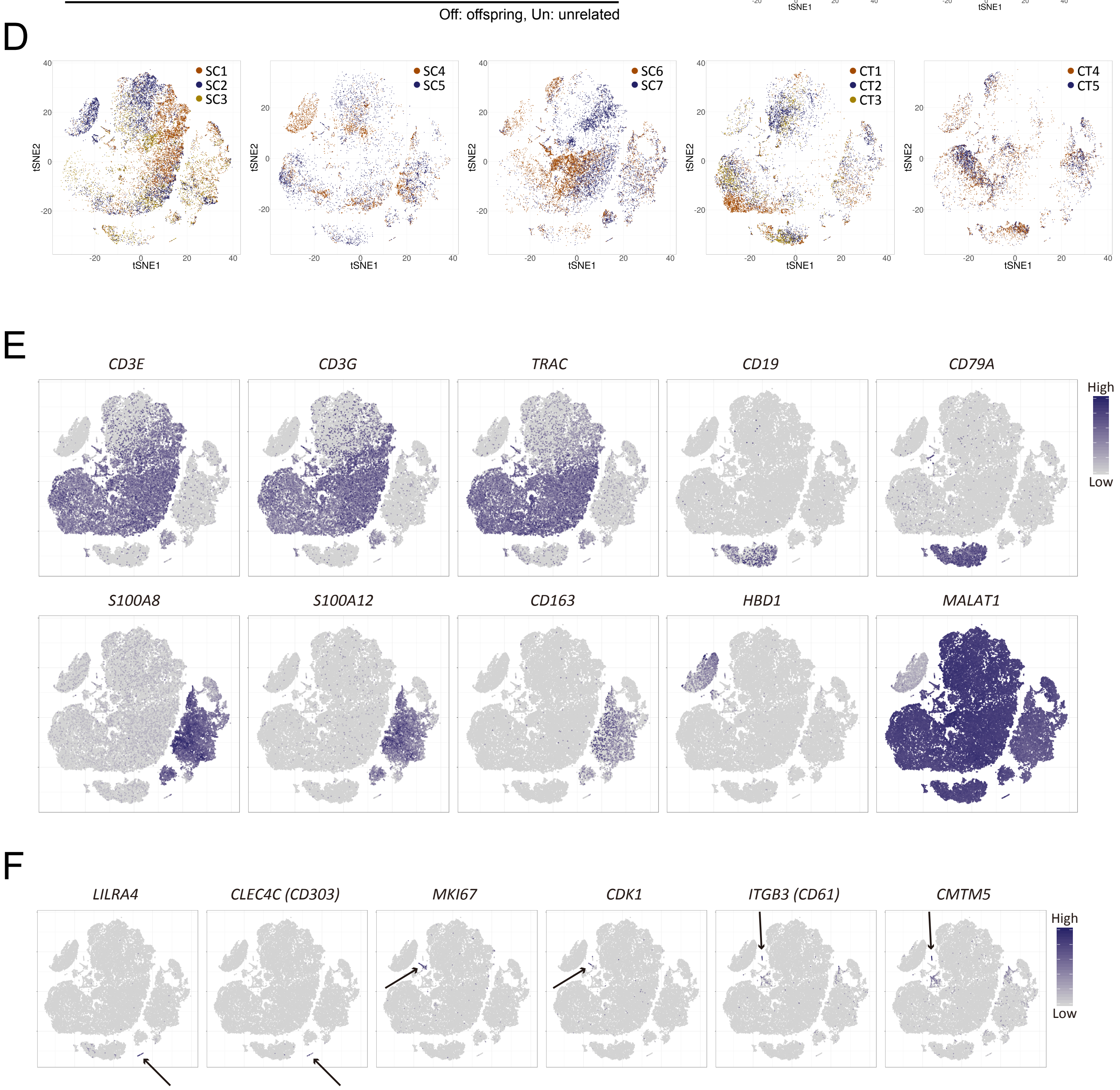

Figure S2

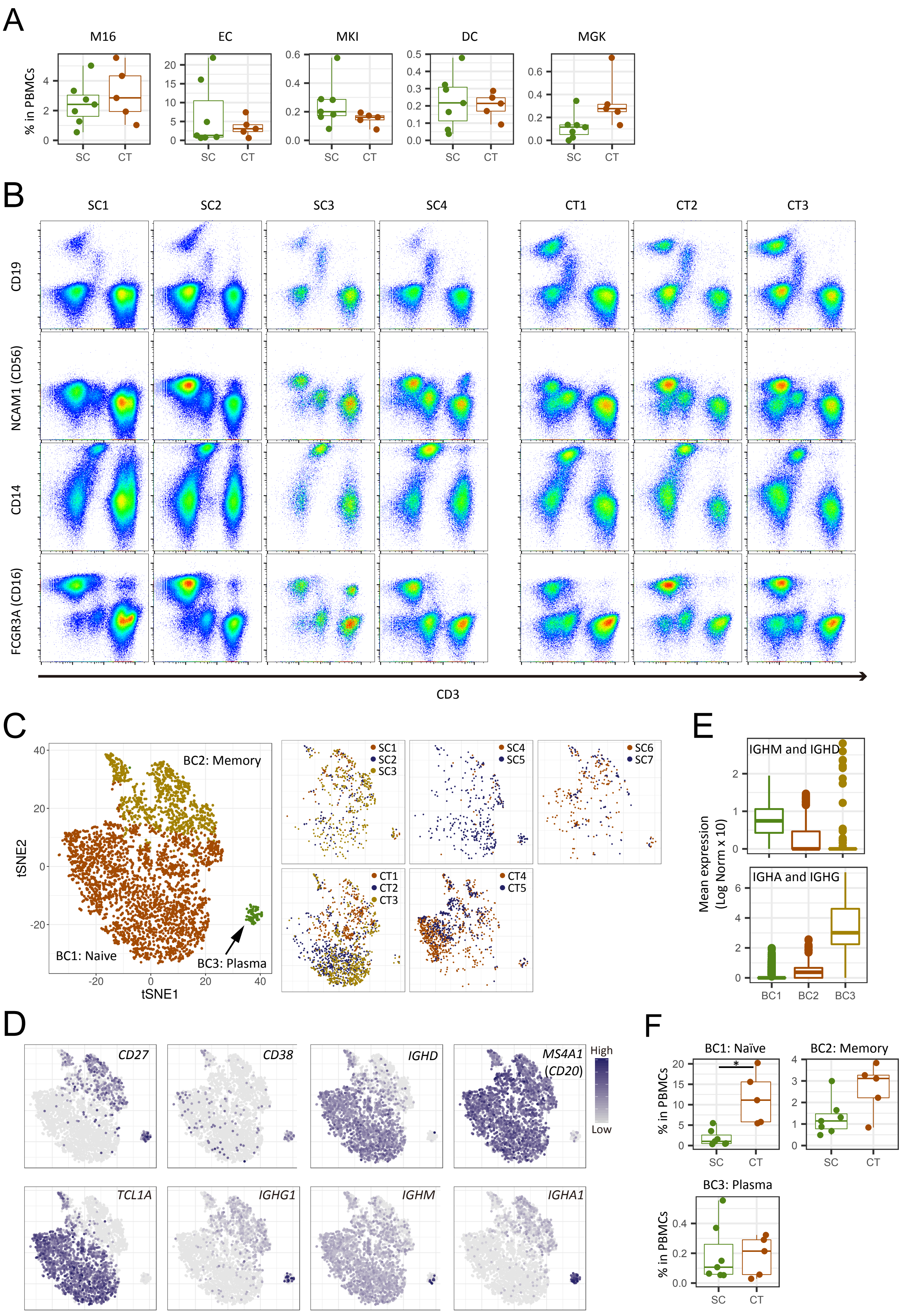

A
M16
EC
MKI
DC
MGK
% in PBMCs
SC
CT
B
SC1
SC2
SC3
SC4
CT1
CT2
CT3
CD19
NCAM1 (CD56)
CD14
FCGR3A (CD16)
CD3
C
BC2: Memory
BC1: Naive
BC3: Plasma
tSNE2
tSNE1
SC1
SC2
SC3
SC4
SC5
SC6
SC7
CT1
CT2
CT3
CT4
CT5
D
CD27
CD38
IGHD
MS4A1 (CD20)
High
Low
TCL1A
IGHG1
IGHM
IGHA1
E
IGHM and IGHD
IGHA and IGHG
Mean expression (Log Norm x 10)
BC1
BC2
BC3
F
BC1: Naïve
BC2: Memory
BC3: Plasma
% in PBMCs
SC
CT

Figure S3

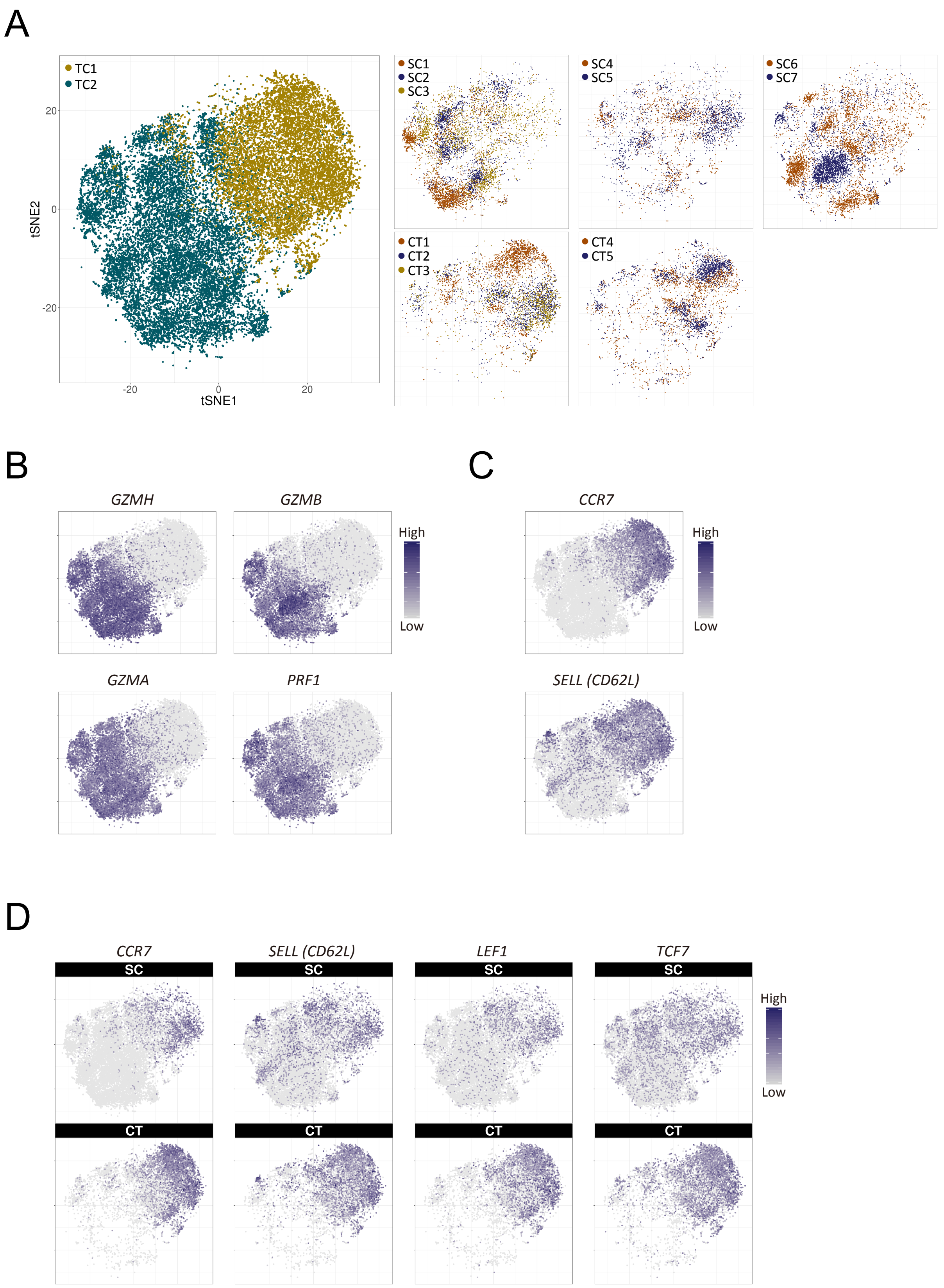
A
TC1
TC2
tSNE2
tSNE1
20
0
-20
SC1
SC2
SC3
SC4
SC5
SC6
SC7
CT1
CT2
CT3
CT4
CT5
B
GZMH
GZMB
GZMA
PRF1
High
Low
C
CCR7
SELL (CD62L)
High
Low
D
CCR7
SELL (CD62L)
LEF1
TCF7
SC
CT
High
Low

Figure S4

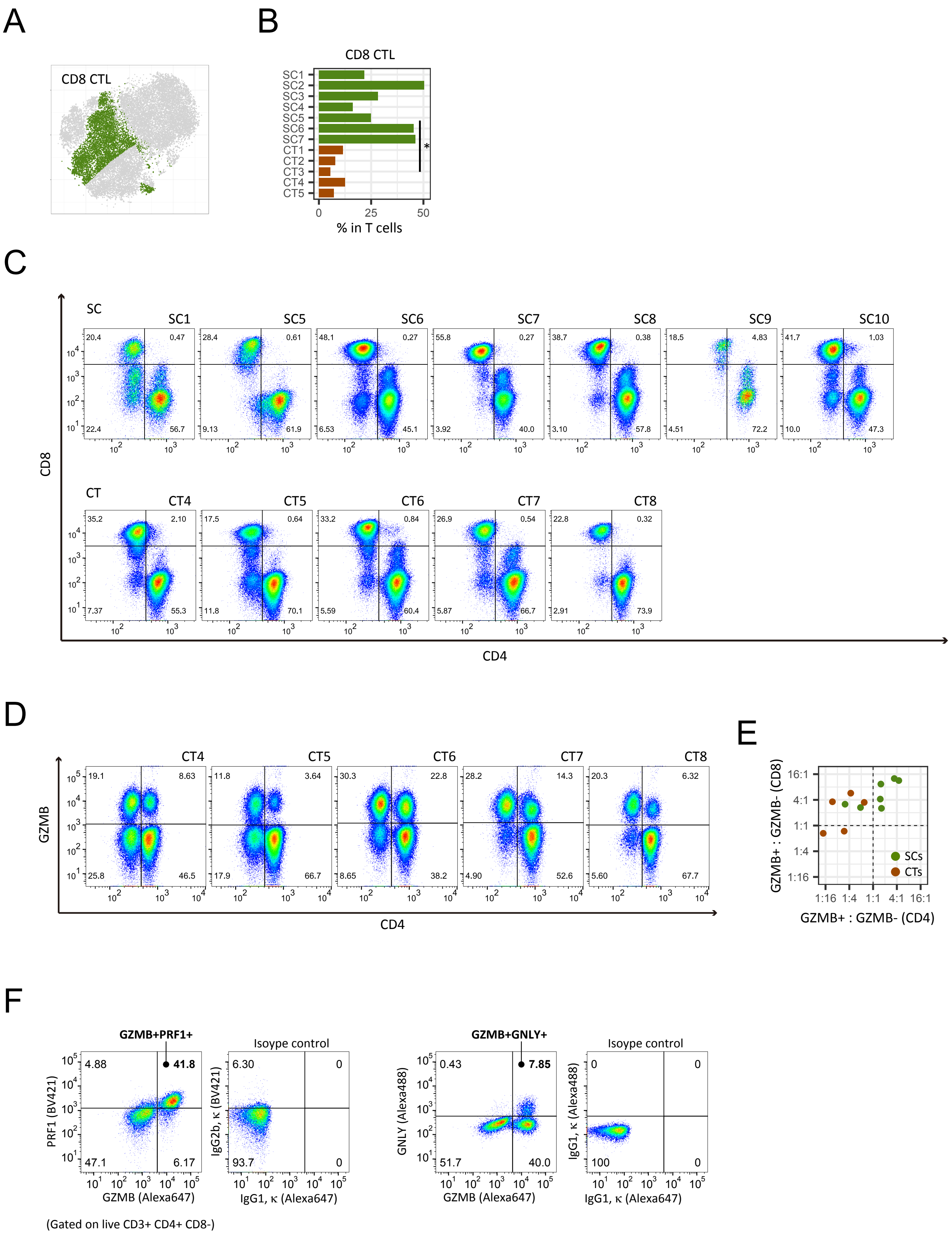

A
CD8 CTL
B
CD8 CTL
SC1
SC2
SC3
SC4
SC5
SC6
SC7
CT1
CT2
CT3
CT4
CT5
*
0
25
50
% in T cells
C
SC
SC1
SC5
SC6
SC7
SC8
SC9
SC10
CT
CT4
CT5
CT6
CT7
CT8
CD8
CD4
D
CT4
CT5
CT6
CT7
CT8
GZMB
CD4
E
GZMB+ : GZMB- (CD8)
GZMB+ : GZMB- (CD4)
16:1
4:1
1:1
1:4
1:16
SCs
CTs
F
GZMB+PRF1+
Isoype control
GZMB+GNLY+
Isoype control
PRF1 (BV421)
GZMB (Alexa647)
IgG2b, κ (BV421)
IgG1, κ (Alexa647)
GNLY (Alexa488)
GZMB (Alexa647)
IgG1, κ (Alexa488)
IgG1, κ (Alexa647)
(Gated on live CD3+ CD4+ CD8-)

Figure S5

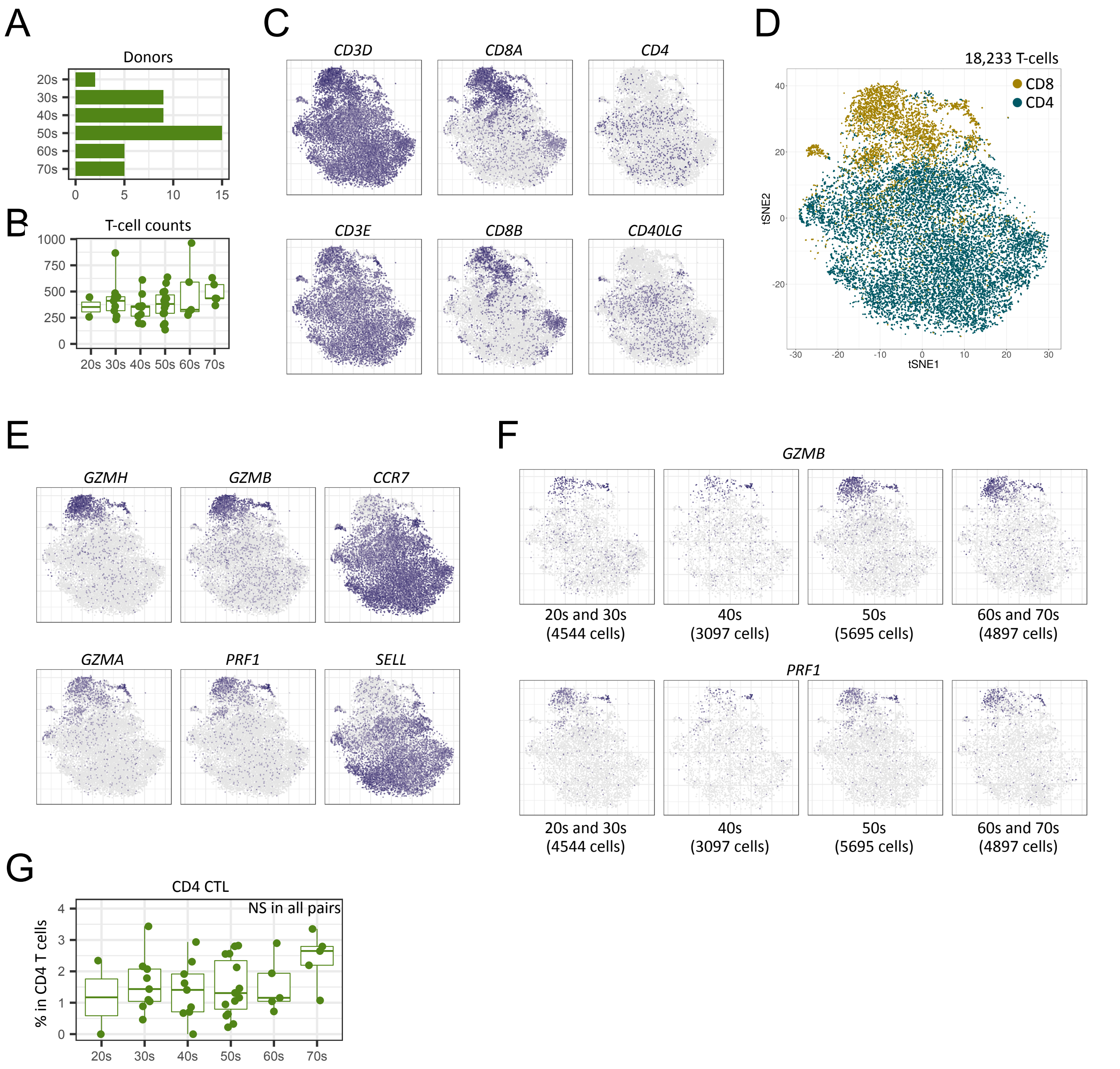

A
Donors
20s
30s
40s
50s
60s
70s
0
5
10
15
B
T-cell counts
1000
750
500
250
0
20s 30s 40s 50s 60s 70s
C
CD3D
CD8A
CD4
CD3E
CD8B
CD40LG
D
18,233 T-cells
CD8
CD4
tSNE2
tSNE1
E
GZMH
GZMB
CCR7
GZMA
PRF1
SELL
F
GZMB
20s and 30s
(4544 cells)
40s
(3097 cells)
50s
(5695 cells)
60s and 70s
(4897 cells)
PRF1
G
CD4 CTL
NS in all pairs
% in CD4 T cells
20s
30s
40s
50s
60s
70s

Figure S6

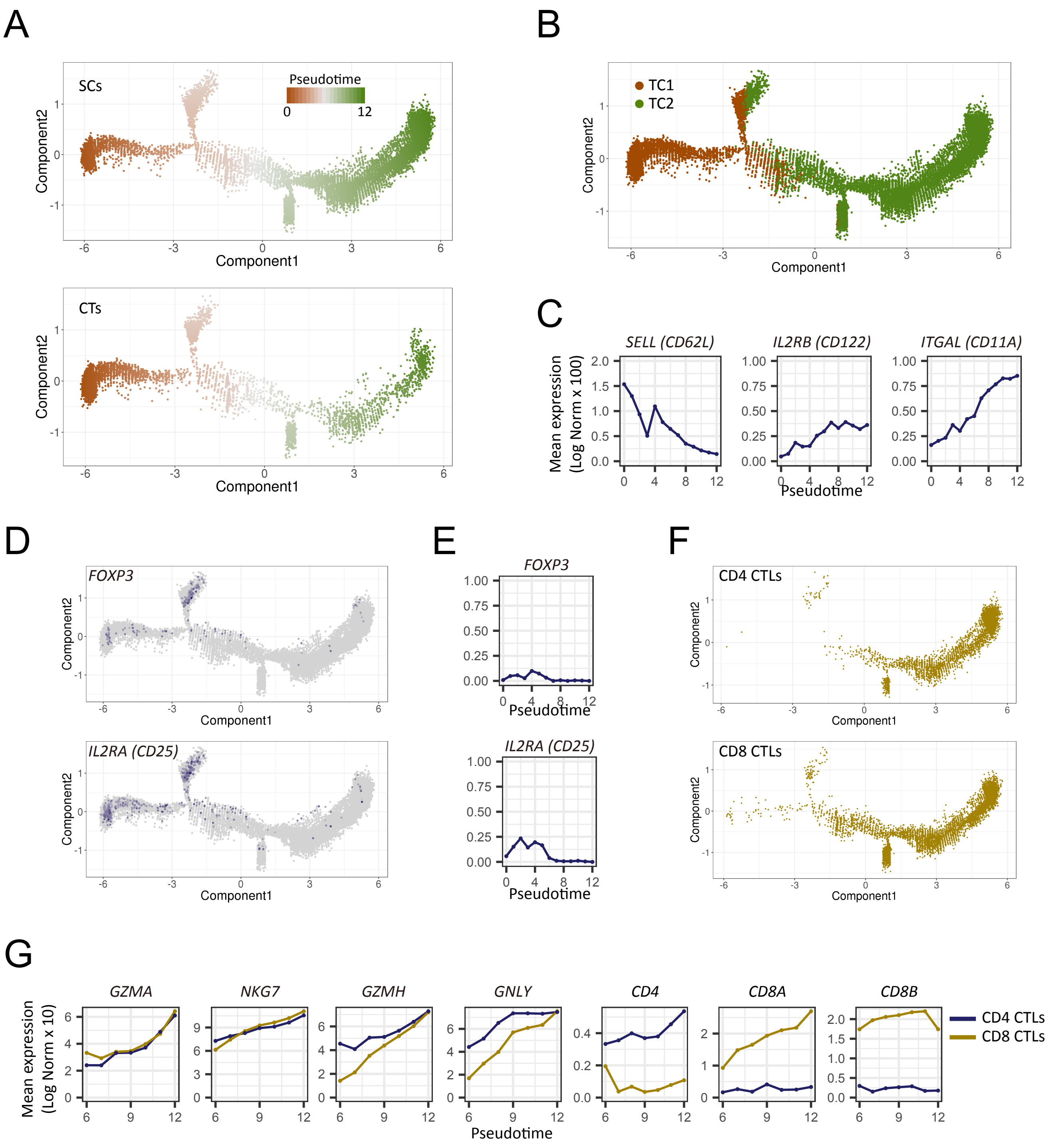
A
SCs
Pseudotime
0
12
Component2
Component1
CTs
B
TC1
TC2
C
SELL (CD62L)
IL2RB (CD122)
ITGAL (CD11A)
Mean expression
(Log Norm x 100)
Pseudotime
D
FOXP3
IL2RA (CD25)
E
F
CD4 CTLs
CD8 CTLs
G
GZMA
NKG7
GZMH
GNLY
CD4
CD8A
CD8B
Mean expression
(Log Norm x 10)
Pseudotime

Figure S7

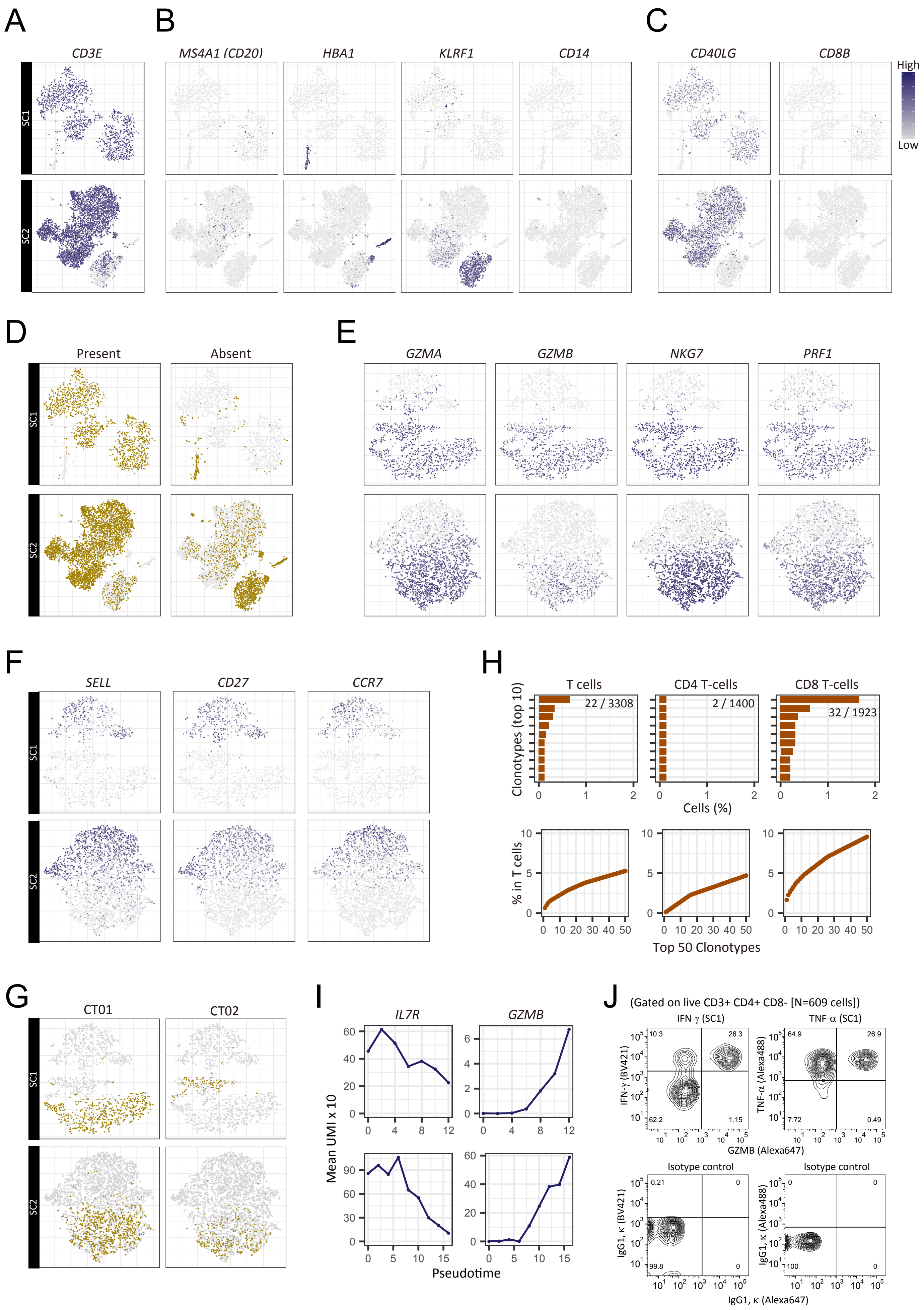

A
CD3E
SC1
SC2
B
MS4A1 (CD20)
HBA1
KLRF1
CD14
C
CD40LG
CD8B
High
Low
D
Present
Absent
E
GZMA
GZMB
NKG7
PRF1
F
SELL
CD27
CCR7
H
T cells
CD4 T-cells
CD8 T-cells
22 / 3308
2 / 1400
32 / 1923
Clonotypes (top 10)
Cells (%)
% in T cells
Top 50 Clonotypes
G
CT01
CT02
I
IL7R
GZMB
Mean UMI x 10
Pseudotime
J
(Gated on live CD3+ CD4+ CD8- [N=609 cells])
IFN-γ (SC1)
TNF-α (SC1)
IFN-γ (BV421)
TNF-α (Alexa488)
GZMB (Alexa647)
Isotype control
IgG1, κ (BV421)
IgG1, κ (Alexa488)
IgG1, κ (Alexa647)

Figure S1. Single-cell transcriptome profile of PBMCs. **a.** Samples used for single-cell RNA-Seq and FACS analyses. FACS1 used antibodies against major cell-type markers: CD3, CD19, CD14, CD16, and NCAM1 (CD56), whereas FACS2 used antibodies against T-cell subtype markers: CD3, CD4, CD8, and GZMB. **b.** Median numbers of UMI counts per cell for each donor **c.** Two-dimensional tSNE visualization, separated into supercentenarians and controls. Different colors in the right panel represent 4 age groups. **d.** Two-dimensional tSNE visualization, separated into two or three donor groups. **e.** Expression of marker genes for cell-type markers and *MALAT1* as the highest expressed gene; cell positions are from the t-SNE plots in Fig. 1c. **f.** Expression of marker genes used to define three small clusters by k-means clustering (i.e., MKI67+ proliferating cells [MKI], dendritic cells [DC], and megakaryocytes [MGK]).

Figure S2. Reduction in the number of B cells in supercentenarians. **a.** Boxplots of the percentage of each indicated cell type in PBMCs. **b.** FACS plots for four supercentenarians (SC1–SC4) and three controls (CT1–CT3) profiled using CD3, CD19, NCAM1 (CD56), CD14, and FCGR3A (CD16). **c.** Two-dimensional tSNE visualization of B cells for all 12 donors or separately for two or three donor groups. **d.** Expression of markers for B-cell subtypes. **e.** Expression of immunoglobulin heavy chains in each B-cell subtype. Upper panel shows total expression of *IGHM* and *IGHD*, which are used before the class switch. Lower panel shows total expression of *IGHA1*, *IGHA2*, *IGHG1*, *IGHG2*, *IGHG3*, and *IGHG4*, which are used after the class switch. **f.** Boxplots of the percentage of each B-cell subtype (defined by k-means clustering) in PBMCs of seven supercentenarians (SC1–SC7) and five controls (CT1–CT5). *, $P < 0.05$ (Wilcoxon rank sum test); no asterisk means not significant.

Figure S3. Expansion of cytotoxic T-cell populations in supercentenarians. **a.** Two-dimensional tSNE visualization of T cells using the Seurat R package. Different colors represent the original TC1 and TC2 clusters; 86.3% of the original TC1 is clustered into Seurat_TC1; 97.6% of the original TC2 is clustered into Seurat_TC2. **b–d.** Expression of cytotoxic genes and lymph node homing markers; cell positions are from the t-SNE plot in **a.**

Figure S4. Expansion of cytotoxic CD4 T-cell populations in supercentenarians. **a.** CD8 CTLs defined based on the expression of *CD8A* and *CD8B*; cell positions are from the t-SNE plot in Fig. 4a. **b.** Percentages of CD8 T-cells among the total T-cells. *, $P < 0.05$ (Wilcoxon rank sum test). **c.** FACS profiles of supercentenarians (SC1, SC5–SC7, SC9, and SC10) and one semi-supercentenarian (SC8), and controls (CT4–CT8). Cells gated on CD3+ were profiled using CD4 (x-axis) and CD8 (y-axis). Cells in lower right and upper left corners are classified as CD4 and CD8 T-cells, respectively. **d.** FACS profiles of five controls (CT4–CT8). Cells gated on CD3+

were profiled using CD4 (x-axis) and GZMB (y-axis). **e.** Ratio between the percentage of GZMB+ and GZMB− cells in CD4 (x-axis) and CD8 (y-axis) T-cells. Ratio “1:1” indicates that the percentage of GZMB+ cells equals that of GZMB− cells. **f.** FACS profiles of one supercentenarian (SC2). Cells gated on live CD3+ CD4+ CD8- were profiled using GZMB or IgG1 κ (x-axis) and PRF1, GNLY, or IgG2b κ (y-axis).

Figure S5. Expressions of cytotoxic genes in T-cells from young donors. **a.** The numbers of donors used in the analysis for each decade of age. **b.** The numbers of T-cells per donor in each decade of age. **c.** Expression of the indicated marker genes. **d.** Two-dimensional tSNE visualization of 18,233 T cells using the Seurat R package. Different colors represent two subtypes defend by the original paper. **e.** Expression of the indicated marker genes. **f.** Expression of *GZMB* and *PRF1* in each age group. **g.** Percentages of estimated CD4 CTLs in CD4 T-cells. NS means not significant (Wilcoxon rank sum test).

Figure S6. The differentiation state of T cells in supercentenarians. **a.** Pseudotime trajectory of T cells, shown separately for supercentenarians (SC) and controls (CT). **b.** Pseudotime trajectory of T cells colored by TC1 and TC2. **c.** Expression transition of differentiation-associated genes. **d.** Expression of *FOXP3* and *IL2RA* (*CD25*) mapped in pseudotime. **e.** Expression transition of *FOXP3* and *IL2RA* (*CD25*) along the pseudotime. **f.** Distribution of CD4 and CD8 CTLs mapped in pseudotime. **g.** Expression transition of selected genes shown separately for CD4 and CD8 CTLs.

Figure S7. Single-cell 5′ transcriptome and TCR profiles for two supercentenarians (SC1, upper panels and SC2, lower panels). **a.** Expression of a marker gene for T-cells. **b.** Expression of marker genes for B-cells (*MS4A1*), erythrocytes (*HBA1*), NK cells (*KLRF1*), and monocytes (*CD14*). **c.** Expression of marker genes for CD4 T-cells (*CD40LG*) and CD8 T-cells (*CD8B*). **d.** Presence or absence of cells in TCR libraries. **e.** Expression of marker genes for cytotoxic T-cells. **f.** Expression of marker genes for T-cell differentiation. **g.** Distribution of the top 2 most abundant clonotypes, CD01 and CT02, on the t-SNE map. **h.** Diversity of TCRs in publicly available datasets released by another group. **i.** Transient expression of genes associated with T-cell differentiation. **j.** FACS profiles of one supercentenarian (SC1) upon PMA/ionomycin stimulation. Cells gated on live CD3+ CD4+ CD8- were profiled using GZMB or IgG1 κ (x-axis) and IFN-γ, TNF-α, or IgG1 κ (y-axis). The graph is shown in contour plot due to the small number of cells (609 cells) available.
